# Mental Health Symptoms during the COVID-19 Pandemic among Cancer Survivors Who Endorse Cannabis: Results from the COVID-19 Cannabis Health Study

**DOI:** 10.3390/curroncol29030170

**Published:** 2022-03-19

**Authors:** Diane L. Rodriguez, Denise C. Vidot, Marlene Camacho-Rivera, Jessica Y. Islam

**Affiliations:** 1 Morsani College of Medicine, University of South Florida, Tampa, FL 33602, USA; dianer@usf.edu; 2 School of Nursing and Health Studies, University of Miami, Coral Gables, FL 33146, USA; dvidot@med.miami.edu; 3 Sylvester Comprehensive Cancer Center, Miller School of Medicine, University of Miami, Miami, FL 33136, USA; 4 Department of Community Health Sciences, School of Public Health, SUNY Downstate Health Sciences University, Brooklyn, NY 11203, USA; marlene.camacho-rivera@downstate.edu; 5 Cancer Epidemiology Program, H. Lee Moffitt Cancer Center and Research Institute, Tampa, FL 33612, USA

**Keywords:** depression, anxiety, mental health, cannabis, COVID-19 pandemic, palliative care

## Abstract

Our objective was to examine the prevalence of mental health symptoms and the behavioral impact of the COVID-19 pandemic on cancer survivors who endorse cannabis. Participants included 158 adults (≥18 years) who self-reported medicinal cannabis use and responded to our internet-based questionnaire (21 March 2020–24 March 2021). Data included 79 cancer survivors and 79 age-matched adults without a history of cancer. Descriptive statistics were used to compare demographics, the prevalence of generalized anxiety (GAD-7), depression (CES-D-10), and changes in behavior during the COVID-19 pandemic by cancer survivorship status. Overall, 60.8% and 48.1% of cancer survivors self-reported the use of cannabis to manage their anxiety and depression, respectively. Probable clinical depression (CES-D-10 score ≥ 10) and anxiety (GAD-7 score ≥ 10) were identified in 50.7% and 38.9% of cancer survivors, respectively. Cancer survivors were more likely to report that their anxiety symptoms made it very or extremely difficult to work, take care of home, or get along with others than their counterparts. Cancer survivors with anxiety and/or depression were more likely to fear giving COVID-19 to someone else (47.5% vs. 23.1%, *p* = 0.023) and to fear being diagnosed with COVID-19 (77.5% vs. 38.5%, *p* < 0.001) compared to cancer survivors without anxiety and depression symptoms. Further research is recommended to evaluate the use of cannabis as palliative care to improve mental health among cancer survivors.

## 1. Introduction

Since the medical use of cannabis was legalized in California in 1996, an increasing number of states in the United States (US) have followed. Currently, the use of medical and/or recreational (adult) cannabis is allowed in 36 states and four territories, including the District of Columbia [1]. These policy changes, along with a decreased risk perception, changes in societal attitudes, and affordability have led to an increasing interest in the use of cannabis for the management of certain symptoms [2,3,4]. Some of the most common reasons for the use of cannabis-based products include the management of pain, nausea/vomiting, and mental health symptoms [4,5]. These are prevalent symptoms in cancer patients and survivors, for whom the use of medical cannabis may represent a potential non-curative therapy [2,6,7,8,9,10]. While data are limited regarding the prevalence of cannabis use among cancer patients, a Canadian study found that 18% of cancer patients surveyed had used cannabis in the last 6 months, and 13% use it for cancer-related symptoms [11]. Further, in the United States, recently published estimates using national data collected between 2013–2018 suggest that 8% of cancer survivors may have used cannabis in the past 12 months [12]. The use of cannabis has further been suggested as a potentially valuable supplement to palliative treatment, especially when symptoms are difficult to treat with other available treatment measures, and when benefits outweigh potential risks [13,14,15].

Cancer patients and survivors experience a significant burden of mental health symptoms, such as anxiety and depression, and are more likely to experience these symptoms compared to the general population [16]. A study of over 10,000 cancer patients found that, across cancer types, approximately 19% and another 22% of patients showed clinical and subclinical symptoms of anxiety, respectively, after a cancer diagnosis. Similarly, approximately 12% and another 16% showed clinical and subclinical symptoms of depression, respectively [17]. Overall, studies have found that approximately one-third of cancer patients are affected by mental health disorders [4,16,18,19]. Poor quality of life due to poor negative mental health outcomes is of concern among cancer patients as prior research suggests that depressed cancer patients can be up to three times less likely to adhere to medical treatment [20]. This is of particular importance for minoritized and underserved populations who are more likely to discontinue medical treatment or experience barriers to treatment completion [21], and already bear the disproportionate burden of poorer health outcomes compared to their White counterparts [22]. When poorly managed or left unrecognized, anxiety and depression can lead to reduced quality of life and shorter survival [23,24]. Timely and proper management of such symptoms is therefore imperative for the well-being of cancer patients.

Unfortunately, the COVID-19 pandemic has led to a deterioration of mental health among many populations, including those with cancer [25,26]. Per the US Census Bureau, the prevalence of anxiety and depression among US adults was three times higher during the pandemic compared to the prior calendar year [25]. Similar to what we observe pre-pandemic, anxiety and depressive symptoms were more likely to be reported by cancer survivors compared to non-cancer patients during the COVID-19 pandemic [27]. Furthermore, the high prevalence of mental health problems is accompanied by reported gaps in mental health services for cancer patients during the pandemic [28]. 

Despite the prevalence of mental health symptoms and the potential benefits of medicinal cannabis for symptom management, data on the use of cannabis among cancer patients are limited. In this study, we examine the prevalence of mental health symptoms and the behavioral impact of the COVID-19 pandemic on cancer survivors who endorse cannabis compared to adults without a history of cancer.

## 2. Materials and Methods

### 2.1. COVID-19 Cannabis Health Study

The COVID-19 Cannabis Health Study is an internet-based survey designed to examine the impacts of the COVID-19 pandemic on cannabis use patterns and related behaviors among adults who use cannabis for medicinal and/or recreational purposes [29]. The survey included questions regarding health conditions and symptoms managed by cannabis users; measures of mental health symptoms reported since the COVID-19 pandemic, including validated scales to measure clinical anxiety and depression; measures of changes in behavior and stress that may have been experienced by individuals during the pandemic period; and mechanisms employed to cope with the pandemic. Eligibility criteria for the study included adults 18 years of age or older who self-identified as a cannabis user. Participants were recruited using a convenience sampling approach via social media, electronic mail, study flyers, and other networks digitally. Further details are provided in a prior publication [29]. For our analysis, data only include responses from participants who reported using cannabis to manage a chronic health condition.

This study was approved by the University of Miami Institutional Review Board. Informed consent was provided by all participants electronically before beginning the survey. REDCap software, hosted at the University of Miami, was used for data collection and management. Overall, we received 3,594 responses. For this study, data included responses from 160 participants including 80 participants who indicated use of cannabis to manage cancer as a chronic health condition (cancer survivors) (3.2%) along with age and sex-matched medicinal cannabis users without a history of cancer (*n* = 80). To carry out the age matching we created age groups including 18–24, 25–34, 35–44, 45–54, 55–64, and 65+ years. We matched respondents based on these age groupings. Responses were received from 21 March 2020 to 23 March 2021.

### 2.2. Measures

The primary exposure for this analysis was cancer survivorship status. To define cancer survivor, we used the U.S. National Cancer Institute’s definition: “a person with cancer from the time of diagnosis until the end of life.” Responses to the following two questions within the study were used to categorize participants as cancer survivors: “Which of the following conditions do you currently live with?” or “What conditions do you manage with your cannabis?”. Participants were classified as cancer survivors if they selected “cancer” as a response to either of those two questions. 

Anxiety symptoms were measured by the responses to the 7-item Generalized Anxiety Disorder Scale (GAD-7) questionnaire included within the COVID-19 Health Study Survey [30]. The GAD-7 questionnaire is designed to evaluate for the presence of generalized anxiety disorder. Participants rate their frequency of symptoms experienced within the 2 weeks prior to the interview using a 4-point scale (0 = ‘not at all’; 1 = ‘several days’; 2 = ‘over half the days’; 3 = ‘nearly every day’). A participant’s score can then range from 0 to 21. Higher scores indicate worse anxiety symptoms. A score of 10 or greater was used as the cut-off for the indication of a generalized anxiety disorder [30]. 

Similarly, the 10-item Center for Epidemiologic Studies Depression Scale (CESD-10) questionnaire was used to measure depressive symptoms among respondents [31]. It contains 10 questions about symptoms that occurred in the week prior to the interview. Participants rate their frequency of symptoms also on a 4-point scale (from 0 = ‘rarely or none of the time’ to 3 = ‘most or all of the time’). A participant’s score can then range from 0 to 30. Higher scores also suggest a greater severity of depressive symptoms. A score of 10 or greater was used as the cut-off for the indication of depression [32]. 

The revised Pandemic Stress Index (PSI) is a three-item measure of changes in behavior and stress that may have been experienced by individuals during the COVID-19 pandemic. It was used to evaluate the impact of the pandemic on the participants’ daily lives [33]. The survey included the questions: “What are you doing/did you do during the COVID-19 pandemic?” which assesses behavior changes in response to COVID-19, including changes in response to public health messaging, changes in the workplace, and changes to protect one’s own or others’ health, “How much is/did the COVID-19 pandemic impact your day-to-day life?” which uses a 5-point scale, and “Which of the following are you experiencing (or did you experience) during the COVID-19 pandemic?” which is a multichoice checklist of items that pertain to emotional distress, stigma, financial stress, sexual behavior, and support [34].

The COVID-19 Cannabis Health Questionnaire also included questions about behaviors used to cope with the COVID-19 pandemic including mediation, eating or physical activity, sleep habits, talking to friends and family or health care professionals, cannabis use, and sexual activity [35]. Coping behaviors are defined as conscious or voluntary acts to manage stressful situations. Details of the COVID-19 Cannabis Health Questionnaire are located in RTI International’s PhenX Toolkit (https://www.phenxtoolkit.org/covid19/, accessed on 12 December 2021), a catalog of recommended measurement protocols.

### 2.3. Data Analysis

Chi-squared or Fisher’s Exact test (when appropriate) tests among the age and sex-matched sample were used for univariate comparisons of categorical variables including demographic characteristics, measures of anxiety and depression, behavior changes, and stress experienced during the COVID-19 pandemic, and coping behaviors by cancer survivorship status. We used *t*-tests to compare continuous variables such as the CESD-10 score. We used a complete case approach due to the limited missing value (<10%). Statistical analysis was performed using SAS v9.4 (SAS Institute, Cary, NC). The Type I error was maintained at 5%.

## 3. Results

### 3.1. Demographics

Most participants reported living in the United States (88%), while 12% of participants reported living in other countries including Canada, Colombia, France, Israel, Kenya, Malaysia, Mexico, and New Zealand. The mean age, median age, and age range of respondents were 57.8, 60, and 29–86 years, respectively. Racial distribution, as self-reported, was 82.2% White, 8.3% Hispanic, 3.8% Black or African American, 1.9% Asian, and 3.8% Other. Overall, 61.3% and 48.8% of cancer survivors self-reported using medicinal cannabis to manage their anxiety and depression, respectively. Additionally, 55.0% of cancer survivors reported using cannabis to manage their chronic pain. (Table 1).

### 3.2. Assessment of Anxiety and Depression

With GAD-7 scores at or above 10, 38.3% of cancer survivors and 31.3% of non-cancer patients reported anxiety (Table 2). Table 3 describes the frequency of participants’ answers on each of the CES-D-10 items as reported by cancer survivors and those without a history of cancer, the frequency of participant’s total scores, and the frequency of scores above and below the cut-off for depression indication. With CES-D-10 scores at or above 10, 50.7% of cancer survivors and 53.3% of adults without a history of cancer reported depression. No statistically significant differences were observed by cancer survivor status on overall anxiety and depression measures.

### 3.3. Behavioral Impacts of the COVID-19 Pandemic

Table 4 describes the impact of the COVID-19 pandemic on behaviors among cancer survivors with probable anxiety or depression (as defined by a score of 10 or above on either the GAD-7 or CES-D-10 questionnaires) (*n* = 41; 51.3%) and those with no mental health condition. Cancer survivors with anxiety or depression reported to be more likely to fear giving COVID-19 to someone else (46.3% vs. 23.1%, *p* = 0.029) and to fear being diagnosed with COVID-19 (78.0% vs. 38.5%, *p* < 0.001) compared to cancer survivors without mental health conditions. 

Cancer survivors with anxiety or depression were more likely to report that the pandemic has impacted their daily life “very much” compared to those without these mental health outcomes (58.5% vs. 20.5%, *p* < 0.001). The main pandemic-related coping mechanisms reported by cancer survivors with anxiety or depression included more sleep (48.8%), practicing meditation/mindfulness (46.3%), physical activity (46.3%), talking to family and friends (43.9%), overeating or stress-eating (24.4%), and more sex (24.4%). 

When asked the question “What are you doing/did you do during the COVID-19 pandemic?”, cancer survivors with probable anxiety or depression were more likely to report practicing social distancing as prevention (90.2% vs 61.5%, *p* = 0.003) and to report following media coverage (63.4% vs 30.8%, *p* = 0.003). When asked to describe their experiences during the COVID-19 pandemic, cancer patients with probable anxiety or depression were also more likely to report worrying about local friends, family, and/or partners (75.6% vs 51.3%, *p* = 0.024), experiencing financial loss (58.5% vs 35.9%, *p* = 0.043), experiencing frustration or boredom (58.5% vs 28.2%, *p*= 0.006), not having enough basic supplies such as water or medication (41.5% vs 12.8%, *p* = 0.004), and getting less sleep (24.4% vs 5.1%, *p* = 0.016) than cancer patients with no mental health condition.

## 4. Discussion

This study describes the prevalence of anxiety and depressive symptoms, changes in behavior experienced by cancer survivors during the pandemic period, and the mechanisms employed to cope with the COVID-19 pandemic among an age-matched sample of respondents from the COVID-19 Cannabis Health Study. The results from this study suggest that medicinal cannabis users with and without cancer frequently use cannabis to manage their mental health symptoms (see Table 1). Overall, 60.8% and 48.1% of cancer survivors self-reported medicinal cannabis use to manage their anxiety and depression, respectively. Feelings of anxiety and depression, however, differ from anxiety and depression as mental health disorders, which involve excessive fear/anxiety or depressive symptoms that interfere with daily activities or important areas of functioning, are associated with a minimum number of certain symptoms, and prevail for a certain minimum amount of time. The criteria for each clinical anxiety and depression disorders are defined in the fifth edition of the Diagnostic and Statistical Manual of Mental Disorders (DSM-5). As such, using the results of the GAD-7 and CES-D-10 questionnaires, probable clinical anxiety and depression were identified in 38.9% and 50.7% of cancer survivors, respectively, with no statistically significant differences observed between cancer and non-cancer patients (see Table 2 and Table 3). We found that the COVID-19 pandemic is impacting cancer survivors who endorse cannabis, particularly those with anxiety and/or depression. Cancer survivors with anxiety or depression were more likely to fear giving COVID-19 to someone else, fear being diagnosed with COVID-19 themselves, and also more likely to report that their life has been highly impacted by the pandemic. Our findings demonstrate the significance of addressing negative mental health outcomes during the COVID-19 pandemic, particularly among vulnerable populations such as cancer patients who endorse medicinal cannabis. 

Overall, over half of cancer survivors reported they use medicinal cannabis to manage their anxiety and/or depression. This compares to approximately 34.3% of the general adult population in the US with either anxiety or depression or both during the pandemic period, as reported by the US Census Bureau [25]. The higher prevalence of mental health symptoms among cancer survivors in our sample may be explained in part by the burden of cancer, including physical, emotional, and financial burden, as demonstrated in prior studies [37,38,39]. The difference, however, may also be reflective of the characteristics of the population in our sample, as one of the main reasons for the use of cannabis is the management of mental health symptoms. Similarly, approximately 41.8% and 67.1% of respondents without a cancer history reported using cannabis for the management of anxiety and depression symptoms, respectively. The second most prevalent reason for the use of cannabis was for the management of chronic pain, reported by 54.4% and 49.4% of cancer and non-cancer patients, respectively. Other symptoms managed by the use of cannabis by respondents included post-traumatic stress disorder, irritable bowel syndrome symptoms, and another autoimmune disease. 

Overall, symptoms of depression were found to be more prevalent than symptoms of anxiety in this study sample. However, anxiety symptoms were also found to make work and household activities, as well as relationships with other people, more difficult for cancer patients than for adults without a history of cancer. This adds to the changes and challenges cancer patients already typically endure with respect to abilities and independence [40]. Depression in cancer has also been correlated with lower patient survival, with higher levels of depressive symptoms predicting higher mortality [41]. Cancer patients who experience mental health symptoms are less likely to adhere to their recommended medical curative treatment as well as survivorship care [20]. This is especially important in populations where adherence to treatment may also be impacted by other reasons, including socioeconomic factors. Unmet socioeconomic needs, for example, can lead to missed chemotherapy or radiation appointments [21]. As such, cancer disparities and poorer outcomes are associated with low socioeconomic status and are more prevalent in minority racial and ethnic groups [42]. Medicinal cannabis can be leveraged to close the gap in these observed disparities in the context of palliative care due to several factors such as access and potentially fewer drug interactions with curative cancer treatment. 

Mental health symptoms are also correlated with lower quality of life, particularly when left unrecognized or poorly managed [23,24]. Symptom management and quality of life are especially important in the palliative care setting. Pain, loss of appetite, nausea, vomiting, insomnia, depression, and anxiety are some of the main symptoms targeted by palliative care [43]. These overlap with the symptoms targeted using cannabis. Thus, cannabis represents a treatment opportunity, as a complement to therapy, or when other treatments are inaccessible or have failed to provide relief, and when the benefits outweigh the risks [13,14,15]. Cancer-associated stressors, besides the psychological impacts of diagnosis and fears associated with prognosis and disease recurrence, often include a financial aspect as well [44]. The cost of treatment, changes in or loss of job, and insurance considerations represent an additional burden for cancer patients. In accordance, results from our study also found that cancer patients were more likely to not be working before the COVID-19 pandemic. They were also more likely to report obtaining financial support than adults without a history of cancer (16.5% vs. 5.1%, *p* = 0.021). The affordability of medicinal cannabis, relative to existing cancer treatment costs, presents an important opportunity to reduce the potential additional burden associated with palliative care cancer that patients may experience [9,44,45]. 

The COVID-19 pandemic, besides leading to worsened mental health symptoms across the general population, including cancer patients, has impacted people’s lives in many ways. Our study shows that more than half of cancer survivors reported that the pandemic had significantly impacted their day-to-day life and that this was more significant for cancer survivors with probable anxiety or depression. Main behavior changes among cancer survivors during the pandemic included practicing social distancing as prevention, having a health professional order quarantine, following media coverage, and changing travel plans. Main stressors included worrying about local friends, family, and/or partners, experiencing financial loss, experiencing frustration and/or boredom, and not having enough basic supplies such as water or medication. The main mechanisms for coping with the pandemic included more sleep, talking to family and friends, practicing meditation/mindfulness, engaging in physical activity, overeating or stress-eating, and using more cannabis. It is important to note that while coping mechanisms are employed to manage stressful situations, whether the coping mechanism is adaptive or maladaptive depends on the individual. Our results also indicate that for about 1 in 4 cancer patients who endorse cannabis and experience anxiety and/or depression symptoms, the use of cannabis increased as well. Future research investigating the downstream effects of the negative mental health impacts due to the COVID-19 pandemic on overall survival among cancer patients should be prioritized in the United States, particularly in the context of disparities and socioeconomic vulnerabilities. 

The limitations of this study should be considered when interpreting our presented results. First, data on type of cancer, cancer treatment status, and years since cancer diagnosis were unavailable as the focus of the present study was to evaluate the impact of the pandemic on cannabis users generally. Our research group is investigating the use of medicinal cannabis among cancer patients undergoing active treatment and we will be able to address these limitations in our future work. Due to the anonymity of responses, data may include repeat responses. However, data cleaning and reCAPTCHA methods in REDCap were used to avoid multiple responses and no monetary or other incentives were provided in order to reduce the likelihood of intentional repeat responses. In addition, the generalizability of our findings may be limited. Our small sample size may not fully represent the population of cancer survivors who endorse cannabis; therefore, it limits the generalizability of our results. Internet access was also necessary to respond to our survey. A flyer with the survey link and study details was distributed via social media platforms and emailed to research list serves, community advisory boards, and clinic representatives to share with their networks digitally. Thus, cannabis users without internet access were less likely to be included in our study sample. Furthermore, recall bias and misclassification bias may exist as all data were self-reported. Medicinal cannabis use was self-report as well and was not confirmed via medical record or prescription. Due to mortality rates and inclusion of only cancer survivors, survivorship bias should also be considered when interpreting our study results. 

## 5. Conclusions

Overall, we observed that cancer survivors who endorse cannabis frequently use cannabis to manage their mental health symptoms, as well as chronic pain. While cancer patients commonly report using cannabis for the management of these mental health symptoms, anxiety and depression are not current indications for medicinal cannabis in most states where medicinal cannabis is legalized. Further research is then needed to understand its effectiveness and application for the management of anxiety and depression, its potential adverse effects, and to evaluate its use in the palliative care context to improve mental health and quality of life among cancer patients. Given the prevalence of mental health symptoms in the population of cancer patients who endorse cannabis and the use of cannabis for targeting such symptoms, the need for further research to understand the effectiveness and applications, as well as its potential adverse effects, of medical cannabis is a critical area of research among cancer survivors.

## Figures and Tables

**Table 1:** Demographic characteristics of COVID-19 Cannabis Health Questionnaire Respondents (21 March 2020 to 23 March 2021) among age and sex-matched cancer survivors (*n* = 160) [36].

Characteristic	Total		Adults without Cancer Who Endorse Cannabis		Cancer Survivors Who Endorse Cannabis		*p*-Value
	*n*	%	*n*	%	*n*	%	
Do you live in the United States?							0.807
No	19	11.9	9	11.3	10	12.5	
Yes	141	88.1	71	88.8	70	87.5	
Education							0.923
High school or less	17	10.6	8	10	9	11.3	
Bachelor’s degree or some college	99	61.9	49	61.3	50	62.5	
Master’s degree or higher	44	27.5	23	28.7	21	26.3	
Household Income ^a^							0.584
Less than USD 30,000	34	29.1	15	25.4	19	32.8	
Between USD 30,000 and USD 50,000	30	25.6	17	28.8	13	22.4	
Between USD 50,000 and USD 100,000	27	23.1	12	20.3	15	25.9	
More than USD 100,000	26	22.2	15	25.4	11	19	
Race/Ethnicity ^a^							0.783
Non-Hispanic White	129	82.2	65	82.3	64	82.1	
Non-Hispanic Black or African American	6	3.8	2	2.5	4	5.1	
Hispanic	13	8.3	6	7.6	7	9	
Asian	3	1.9	2	2.5	1	1.3	
Other	6	3.8	4	5.1	2	2.6	
Conditions Managed Using Cannabis ^b^							
None	10	6.3	10	12.7	0	0	0.001
Anxiety	98	61.3	49	61.3	49	61.3	1.000
Depression	74	46.3	35	43.8	39	48.8	0.526
Post-traumatic stress disorder	38	23.8	18	22.5	20	25.0	0.710
HIV/AIDS	3	1.9	0	0	3	3.8	0.080
Chronic Pain	89	55.6	45	56.3	44	55	0.874
Other Autoimmune Disease	17	10.6	7	8.8	10	12.5	0.442
Multiple sclerosis, lateral sclerosis, spinal cord injury	6	3.8	3	3.8	3	3.8	1.000
Crohn’s disease	4	2.5	2	2.5	2	2.5	1.000
Irritable bowel syndrome	21	13.1	9	11.3	12	15.0	0.482

^a^ Does not include a response from all participants, as participants were allowed to choose to not respond to this question. Missing: Income (*n* = 43), race/ethnicity (*n* = 3). ^b^ Participants could choose more than one option.

**Table 2:** Components of the GAD-7 questionnaire among cancer survivors and adults without cancer (*n* = 160).

Characteristic	Total		Adults without Cancer Who Use Cannabis		Cancer Survivors Who Use Cannabis		*p*-Value
	No.	Col %	No.	Col %	No.	Col %	
Felling nervous, anxious, or on edge							0.342
Not at all	51	34.5	27	38	24	31.2	
Several days	38	25.7	21	29.6	17	22.1	
Over half the days	24	16.2	10	14.1	14	18.2	
Nearly every day	35	23.6	13	18.3	22	28.6	
Worrying too much about different things							0.733
Not at all	52	35.6	26	37.7	26	33.8	
Several days	45	30.8	23	33.3	22	28.6	
Over half the days	18	12.3	7	10.1	11	14.3	
Nearly every day	31	21.2	13	18.8	18	23.4	
Trouble relaxing							0.269
Not at all	60	40.8	34	47.9	26	34.2	
Several days	37	25.2	18	25.4	19	25	
Over half the days	20	13.6	7	9.9	13	17.1	
Nearly every day	30	20.4	12	16.9	18	23.7	
Being so restless that it’s hard to sit still							0.744
Not at all	77	52.0	40	56.3	37	48.1	
Several days	35	23.6	15	21.1	20	26	
Over half the days	15	10.1	6	8.5	9	11.7	
Nearly every day	21	14.2	10	14.1	11	14.3	
Becoming easily annoyed or irritable							0.290
Not at all	66	44.9	36	51.4	30	39	
Several days	41	27.9	15	21.4	26	33.8	
Over half the days	17	11.6	7	10	10	13	
Nearly every day	23	15.6	12	17.1	11	14.3	
Feeling afraid as if something awful might happen							0.276
Not at all	59	40.4	33	47.1	26	34.2	
Several days	48	32.9	23	32.9	25	32.9	
Over half the days	18	12.3	6	8.6	12	15.8	
Nearly every day	21	14.4	8	11.4	13	17.1	
If you checked off any problems, how difficult have these made it for you to do your work, take care of things at home, or get along with other people?							0.156
Not difficult at all	60	42.0	34	50.0	26	34.7	
Somewhat difficult	56	39.2	24	35.3	32	42.7	
Very difficult	20	14	6	8.8	14	18.7	
Extremely difficult	7	4.9	4	5.9	3	4.0	
Median Composite GAD-7 Scores	6 (2–13)		5 (1–11.5)		7 (3–14)		0.152
Total GAD-7 scores							0.179
Less than 10	89	64.9	44	68.8	45	61.6	
More than or equal to 10	48	35.0	20	31.3	28	38.4	

Missing: Felling nervous (*n* = 12), worrying (*n* = 14), trouble relaxing (*n* = 13), restless (*n* = 12), easily annoyed (*n* = 13), feeling afraid (*n* = 14), difficulty to do work, etc. (*n* = 17), composite and total GAD7 score (*n* = 23).

**Table 3:** Components of the CES-D-10 questionnaire among cancer survivors and adults without cancer (*n* = 160).

Characteristic	Total		Adults Without Cancer who Use Cannabis		Cancer Survivors who Use Cannabis		*p*-Value
	No.	Col %	No.	Col %	No.	Col %	
I was bothered by things that don’t usually bother me							0.499
Rarely or none of the time (less than 1 day)	68	45.9	34	47.2	34	44.7	
Some or a little of the time (1–2 days)	47	31.8	23	31.9	24	31.6	
Occasionally or a moderate amount of the time (3–4 days)	22	14.9	8	11.1	14	18.4	
Most or all of the time (5–7 days)	11	7.4	7	9.7	4	5.3	
I had trouble keeping my mind on what I was doing							0.361
Rarely or none of the time (less than 1 day)	51	34.5	26	36.1	25	32.9	
Some or a little of the time (1–2 days)	40	27	23	31.9	17	22.4	
Occasionally or a moderate amount of the time (3–4 days)	37	25	14	19.4	23	30.3	
Most or all of the time (5–7 days)	20	13.5	9	12.5	11	14.5	
I felt depressed							0.722
Rarely or none of the time (less than 1 day)	63	42.6	28	38.9	35	46.1	
Some or a little of the time (1–2 days)	40	27	21	29.2	19	25	
Occasionally or a moderate amount of the time (3–4 days)	25	16.9	14	19.4	11	14.5	
Most or all of the time (5–7 days)	20	13.5	9	12.5	11	14.5	
I felt everything I did was an effort							0.059
Rarely or none of the time (less than 1 day)	61	41.2	35	48.6	26	34.2	
Some or a little of the time (1–2 days)	46	31.1	17	23.6	29	38.2	
Occasionally or a moderate amount of the time (3–4 days)	18	12.2	6	8.3	12	15.8	
Most or all of the time (5–7 days)	23	15.5	14	19.4	9	11.8	
I felt hopeful about the future							0.598
Rarely or none of the time (less than 1 day)	53	35.8	28	38.9	25	32.9	
Some or a little of the time (1-2 days)	26	17.6	10	13.9	16	21.1	
Occasionally or a moderate amount of the time (3–4 days)	42	28.4	22	30.6	20	26.3	
Most or all of the time (5–7 days)	27	18.2	12	16.7	15	19.7	
I felt fearful							0.555
Rarely or none of the time (less than 1 day)	59	40.1	32	45.1	27	35.5	
Some or a little of the time (1–2 days)	41	27.9	20	28.2	21	27.6	
Occasionally or a moderate amount of the time (3–4 days)	31	21.1	13	18.3	18	23.7	
Most or all of the time (5–7 days)	16	10.9	6	8.5	10	13.2	
My sleep was restless							0.333
Rarely or none of the time (less than 1 day)	44	29.7	18	25	26	34.2	
Some or a little of the time (1–2 days)	37	25	20	27.8	17	22.4	
Occasionally or a moderate amount of the time (3–4 days)	33	22.3	14	19.4	19	25	
Most or all of the time (5–7 days)	34	23	20	27.8	14	18.4	
I was happy							0.59
Rarely or none of the time (less than 1 day)	59	40.1	28	38.9	31	41.3	
Some or a little of the time (1–2 days)	30	20.4	16	22.2	14	18.7	
Occasionally or a moderate amount of the time (3–4 days)	41	27.9	22	30.6	19	25.3	
Most or all of the time (5–7 days)	17	11.6	6	8.3	11	14.7	
I felt lonely							0.607
Rarely or none of the time (less than 1 day)	79	54.1	35	48.6	44	59.5	
Some or a little of the time (1–2 days)	35	24	19	26.4	16	21.6	
Occasionally or a moderate amount of the time (3–4 days)	22	15.1	12	16.7	10	13.5	
Most or all of the time (5–7 days)	10	6.8	6	8.3	4	5.4	
I could not get “going”							0.297
Rarely or none of the time (less than 1 day)	54	36.7	26	36.1	28	37.3	
Some or a little of the time (1–2 days)	40	27.2	24	33.3	16	21.3	
Occasionally or a moderate amount of the time (3–4 days)	33	22.4	15	20.8	18	24	
Most or all of the time (5–7 days)	20	13.6	7	9.7	13	17.3	
Median CES-D-10 Scores	9 (5–16)		9 (4–16)		10.5 (5–16.5)		0.789
Total CES-D-10 scores							0.557
Less than 10	73	51.1	38	53.5	35	48.6	
More than or equal to 10	70	48.9	33	46.5	37	51.4	

Missing: I was bothered, trouble keeping on my mind, I felt depressed, I felt everything I did was an effort, my sleep was restless (*n* = 12); I felt fearful, I was happy, I could not get going (*n* = 13); I felt lonely (*n* = 14); composite and total score (*n* = 17).

**Table 4:** Impact of the pandemic on behaviors among cancer survivors who endorse cannabis by mental health condition (*n* = 80).

Characteristic	Total		No Mental Health Condition		Anxiety or Depression		*p*-Value
	No.	Col %	No.	Col %	No.	Col %	
Do you fear giving COVID-19 to someone else?							0.029
No	52	65	30	76.9	22	53.7	
Yes	28	35	9	23.1	19	46.3	
Do you fear being diagnosed with COVID-19							<0.001
No	33	41.3	24	61.5	9	22.0	
Yes	47	58.8	15	38.5	32	78.0	
Have you isolated yourself from others due to COVID-19? ^a^							0.058
No	9	11.4	7	18.4	2	4.9	
Yes	70	88.6	31	81.6	39	95.1	
Since COVID-19 has been declared a pandemic, how has the dose of your cannabis use changed? ^a^							0.382
The amount has increased	24	31.2	10	27	14	35	
The amount has decreased	7	9.1	5	13.5	2	5	
The amount has stayed the same	46	59.7	22	59.5	24	60	
Since COVID-19 has been declared a pandemic, are you worried about not being able to pay for your cannabis? ^a^							0.211
No	38	49.4	21	56.8	17	42.5	
Yes	39	50.6	16	43.2	23	57.5	
How much is/did the COVID-19 pandemic impact your day-to-day life? ^a^							
Not at all	3	3.8	2	5.1	1	2.4	0.527
A little	9	11.3	7	17.9	2	4.9	0.064
Somewhat	10	12.5	7	17.9	3	7.3	0.151
Much	15	18.8	7	17.9	8	19.5	0.858
Very Much	32	40	8	20.5	24	58.5	<0.000
Coping Mechanisms during the COVID-19 Pandemic ^b^							
I am not coping	7	8.8	2	5.1	5	12.2	0.264
Meditation/mindfulness	36	45	17	43.6	19	46.3	0.805
Overeating or Stress eating	14	17.5	4	10.3	10	24.4	0.096
Healthier eating habits	20	25	11	28.2	9	22	0.518
Physical activity	32	40	13	33.3	19	46.3	0.235
More sleep	38	47.5	18	46.2	20	48.8	0.814
Less sleep	7	8.8	1	2.6	6	14.6	0.056
Working more	9	11.3	4	10.3	5	12.2	0.784
Talking to family or friends	37	46.3	19	48.7	18	43.9	0.666
Talking to health care professional	7	8.8	4	10.3	3	7.3	0.642
Stopped using cannabis	9	11.3	5	12.8	4	9.8	0.665
Using more cannabis	1	1.3	1	2.6	0	0	0.302
More sex	17	21.3	7	17.9	10	24.4	0.481
Less sex	7	8.8	6	15.4	1	2.4	0.041
What are you doing/did you do during the COVID-19 pandemic? ^b^							
No changes to my life or behavior	6	7.5	5	12.8	1	2.4	0.078
Practicing social distancing as prevention	61	76.3	24	61.5	37	90.2	0.003
Practicing social distancing due to exposure	9	11.3	5	12.8	4	9.8	0.665
Self-quarantine	46	57.5	22	56.4	24	58.5	0.848
Health professional ordered quarantine	5	6.3	1	2.6	4	9.8	0.184
Legal quarantine mandate	6	7.5	1	2.6	5	12.2	0.102
Caring for a child at home	8	10	4	10.3	4	9.8	0.941
Caring for an elderly person at home	5	6.3	2	5.1	3	7.3	0.686
Working from home	23	28.7	11	28.2	12	29.3	0.916
Not working before COVID-19	29	36.3	11	28.2	18	43.9	0.144
Not working due to COVID-19	3	3.8	2	5.1	1	2.4	0.527
Using more healthcare services (e.g., going to urgent care)	2	2.5	0	0	2	4.9	0.162
Following media coverage related to COVID-19	38	47.5	12	30.8	26	63.4	0.003
Changing travel plans	33	41.3	16	41	17	41.5	0.968
Which of the following are you experiencing (or did you experience) during the COVID-19 pandemic? ^b^							
Worrying about local friends, family, partners	51	63.7	20	51.3	31	75.6	0.024
Worrying about international friends, family, partners	21	26.3	8	20.5	13	31.7	0.255
Stigma or discrimination related to COVID-19	10	12.5	3	7.7	7	17.1	0.205
Personal financial loss	38	47.5	14	35.9	24	58.5	0.043
Frustration or boredom	35	43.8	11	28.2	24	58.5	0.006
Not having enough basic supplies (e.g., water, medication)	22	27.5	5	12.8	17	41.5	0.004
More sleep	16	20	8	20.5	8	19.5	0.911
Less sleep	12	15	2	5.1	10	24.4	0.016
Change in sexual activity	9	11.3	5	12.8	4	9.8	0.665
Confusion about what COVID-19 is	10	12.5	3	7.7	7	17.1	0.205
Feeling that I was contributing to the greater good by preventing COVID-19	27	33.8	14	35.9	13	31.7	0.692
Getting emotional or social support	20	25	7	17.9	13	31.7	0.155
Getting financial support	13	16.3	4	10.3	9	22	0.156

^a^ Does not include response from all participants, as participants were allowed to and some chose to not respond to this question. Missing: Have you isolated yourself due to COVID-19 (*n* = 1); how has the dose of your cannabis changed and are you worried about not being able to pay for your cannabis? (*n* = 3); ^b^ Participants could choose more than one option.

## Data Availability

The data presented in this study are available on request from the corresponding author.
